# Vibrational spectroscopic, NMR parameters and electronic properties of three 3-phenylthiophene derivatives via density functional theory

**DOI:** 10.1186/2193-1801-3-701

**Published:** 2014-11-28

**Authors:** Yuan Mei-Rong, Song Yu, Xu Yong-Jin

**Affiliations:** Laboratory of Advanced Energy Storage Materials & devices, Center for Advanced Materials & Biotechnology, Research Institute of Tsinghua University in Shenzhen, Shenzhen, 518057 China; Key Laboratory of Electrochemical Energy Storage Devices, Research Institute of Tsinghua University in Shenzhen, Shenzhen, 518057 China

**Keywords:** Thiophene, DFT, Vibrational spectra, NMR analysis, UV–vis spectra

## Abstract

Quantum chemistry calculations have been performed to compute the optimized geometries, vibrational frequencies, and Mulliken Charges at B3LYP/6-31G(d) and B3LYP/6-311++G(d,p) levels for 3-(4-fluorophenyl)thiophene (FPT), 3-(4-nitrophenyl)thiophene (NPT) and 3-(4-cyanophenyl) thiophene (CPT) in the ground state. In addition, the ^13^C and ^1^H NMR are calculated by B3LYP/6-311++G(d,p) and B3LYP/6-311++G(2d,2p) methods. The singlet electronic excited state properties of the three compounds were investigated using the time-dependent density functional method (TD-DFT) at the B3LYP/6-311++G(d,p)//TD- B3LYP/6-311++G(d,p) level of theory. The influence of the substituted groups on C9 atom is discussed.

## 1. Introduction

Thiophene is one of the most studied heterocycles: it is easy to process, chemically stable, and its synthetic applications have been a constant matter of investigation for many years (Giovanna et al. 2005). π-Conjugated polymers and oligomers based on thiophene building blocks are of immense interest in current research due to their interesting electronic and photophysical properties (Kim et al. 2006; Kline et al. 2006; Patra et al. 2011; Zhang et al. 2011; Marsh et al. 2014; Yumura and Yamashita 2014). Recent literature contains numerous reports on the synthesis and properties of molecular systems having thiophene unit (Zhang et al. 2009; Ustamehmetoglu 2014; Dai et al. 2007; Cho et al. 2012; Patil et al. 2011; Balaji et al. 2011). The electronic properties exhibited by the thiophene and polythiophene derivatives have made them important in organic field effect transistors (OFET) (Yang et al. 2005; Mushrush et al. 2003; Osaka et al. 2007), organic light emitting diodes (OLED) (Cicoira et al. 2006; Lim et al. 2013), solar cells (Hara et al. 2003; Cao et al. 2009; Thomas et al. 2008) and supercapacitors (Sivaraman et al. 2013; Yue et al. 2012; Karthikeyan et al. 2012). The electronic properties of thiophene-based materials can be tuned over a wide range through chemical or architecture modification. It includes different substitution at 2, 3 or 4-position of thiophene molecules.

In most cases, the 2 and 5 positions of thiophene are used for the polymerization. The modification of the molecules for special electronic properties is operated on the 3 and 4-positions (Su et al. 2002; Osaka et al. 1997). Poly(3-phenylthiophene) has represented such a purpose. The introduction of a phenyl group into the backbone of polythiophene stabilizes the conjugated π-bonds system and makes it an attractive low band gap material for the use in supercapacitors (Zhang and Shi 2004). The substitution of fluorophenyl group on β-position of thiophene can improve the thermal stability of corresponding polymer. Poly(3-(4-fluorophenyl)thiophene) has a potential application in type III supercapacitors with improved both p-doping and n-doping performance (Shen et al. 2005; Wei et al. 2006).

Density functional theory (DFT) approaches, especially those using hybrid functional, have evolved to a powerful and very reliable tool, being routinely used for the determination of various molecular properties (Li et al. 2011). B3LYP functional has been shown to provide an excellent compromise between accuracy and computational spectra for molecules of large and medium size (Lu et al. 2013; Yanai et al. 2004). To the best of our knowledge, no theoretical work is done on the 3-phenylthiophene derivatives. Therefore, we made an investigation and studied the structure and spectra of the title compounds using the DFT (B3LYP) method. The aim of our work is to compare the different properties among the three compounds which have different functional groups on the 3-phenylthiophene molecules.

## 2. Computational methods

**Figure 1:**
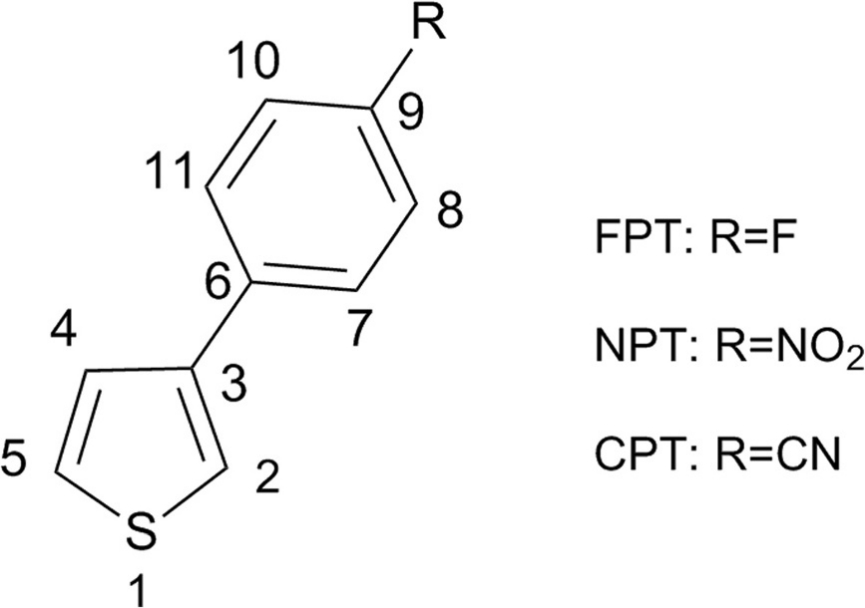
**Molecular structures and atom numbering scheme of the title compounds.** (3-(4-fluorophenyl)thiophene (FPT), 3-(4-nitrophenyl)thiophene (NPT), 3-(4-cyanophenyl)thiophene (CPT)).

All DFT calculations of the title compounds (Figure 1) were carried out using Gaussian09 program package using default thresholds and parameters (Gaussian 09, Revision D.01 et al. 2009). The ground-state structural geometries were fully optimized at the B3LYP method (Yanai et al. 2004) along with the standard 6-31G(d) and 6-311++G(d,p) basis sets. All the parameters were allowed to relax and all the calculations converged to an optimized geometry which corresponds to a true energy minimum revealed by the lack of imaginary frequencies. Vibration frequencies were calculated by using B3LYP/6-31G(d) and B3LYP/6-311++G(d,p) methods (EI-Azhary and Suter 1996). ^1^H and ^13^C NMR chemical shifts are calculated with GIAO approach at B3LYP/6-311++G(d,p) level. The obtained chemical shift values are relative to the shielding of tetramethylsilane (TMS) (Wolff and Ziegler 1998). Time-dependent density functional theory (TD-DFT) (Jacquemin et al. 2009) calculations of electronic spectra were performed on the optimized structure at B3LYP/6-31++G(d,p) levels.

## 3. Results and discussion

### 3.1 Molecular geometry

The optimized geometries of the title compounds have been obtained at B3LYP/6-31G(d) and B3LYP/6-311++G(d,p) levels. Some optimized geometrical parameters are listed in Table 1. To the best of our knowledge, experimental data on the geometric structures of the three title compounds are not available in the literature.

**Table 1:** **Optimized geometrical parameters of the title compounds, bond lengths (Å) and bond angles (** **°** **)**

Parameter	FPT		NPT		CPT	
	6-31G(d)	6-311++G(d,p)	6-31G(d)	6-311++G(d,p)	6-31G(d)	6-311++G(d,p)
S1-C2	1.732	1.730	1.728	1.725	1.728	1.726
C2-C3	1.376	1.374	1.378	1.376	1.378	1.375
C3-C4	1.439	1.436	1.439	1.436	1.439	1.436
C4-C5	1.364	1.363	1.364	1.362	1.364	1.363
C5-S1	1.735	1.732	1.736	1.732	1.735	1.732
C3-C6	1.478	1.478	1.475	1.474	1.475	1.475
C6-C7	1.406	1.403	1.408	1.406	1.407	1.405
C7-C8	1.393	1.392	1.389	1.387	1.389	1.387
C8-C9	1.390	1.386	1.394	1.392	1.405	1.403
C9-C10	1.390	1.385	1.394	1.392	1.405	1.402
C10-C11	1.393	1.392	1.390	1.388	1.389	1.388
C11-C6	1.406	1.403	1.408	1.405	1.407	1.404
C9-F	1.350	1.356	---	---	---	---
C9-N	---	---	1.467	1.474	---	---
N-O1	---	---	1.232	1.226	---	---
N-O2	---	---	1.232	1.226	---	---
C9-C	---	---	---	---	1.433	1.430
C ≡ N	---	---	---	---	1.164	1.156
C5-S1-C2	91.36	91.39	91.44	91.46	91.42	91.45
S1-C2-C3	112.44	112.36	112.38	112.33	112.38	112.32
C2-C3-C4	111.23	111.35	111.33	111.40	111.31	111.40
C3-C4-C5	113.45	113.35.	113.29	113.23	113.32	113.25
C4-C5-S1	111.52	111.55	111.56	111.59	111.56	111.58
C2-C3-C6	124.53	124.43	124.32	124.27	124.35	124.29
C4-C3-C6	124.23	124.22	124.35	124.33	124.33	124.31
C7-C6-C3	121.16	121.11	121.09	121.04	121.14	121.11
C11-C6-C3	120.82	120.81	120.70	120.70	120.78	120.79
C6-C7-C8	121.36	121.11	121.25	121.22	121.24	121.21
C7-C8-C9	118.76	118.59	118.86	118.87	120.04	120.05
C8-C9-C10	121.74	122.07	121.58	121.58	119.37	119.38
C9-C10-C11	118.76	118.59	118.86	118.88	120.05	120.06
C10-C11-C6	121.35	121.33	121.23	121.21	121.22	121.20
C11-C6-C7	118.02	118.08	118.21	118.25	118.08	118.10
C8-C9-F	119.12	118.96	---	---	---	---
C10-C9-F	119.14	118.97	---	---	---	---
C8-C9-N	---	---	119.20	119.21	---	---
C10-C9-N	---	---	119.21	119.21	---	---
C9-N-O1	---	---	117.72	117.73	---	---
C9-N-O2	---	---	117.73	117.73	---	---
C8-C9-C	---	---	---	---	120.31	120.31
C10-C9-C	---	---	---	---	120.32	120.32
C9-C-N	---	---	---	---	179.98	179.98
C2-C3-C6-C7	32.50	35.73	29.50	31.55	30.06	32.59

It is noted from Table 1 that the values of optimized geometrical parameters calculated at B3LYP/6-311++G(d,p) are smaller than that calculated at B3LYP/6-31G(d) level except for the torsion angle of C2-C3-C6-C7. There are little differences on the bond lengths and bond angles among the three title compounds, which indicate that the both the two levels have almost the same calculated accuracy in this system. The C-C bond lengths in benzene ring are between 1.362-1.408 Å, which is much shorter than the typical C-C single bond (1.54 Å) and longer than the C = C double bond (1.34 Å) (Margules et al. 1999). For S1-C2 and S1-C5 bonds, calculated carbon sulfur bond lengths are between 1.725-1.736 Å, which are smaller than the bond length of the single C-S bond (1.82 Å) (Ikawa and Whalley 1996). For FPT, the C9-F bond length is 1.356 Å at B3LYP/6-311++G(d,p) level. The C9-N and C9-C bond lengths are 1.474 Å and 1.430 Å for NPT and CPT, respectively, which stay in the normal range. For NPT, the lengths of the two N-O bonds have almost the same value, which shows a good symmetry within the molecule.

The bond angles C2-S-C5 in the thiophene ring have the value between 91.36°-91.46° for all the three compounds, indicating that the S atom is of sp^2^ hybridization type (Coffman et al. 1996). The bond angles in the benzene rings (120°) have the value between 118°-121° for all the compounds, which may be result from the π delocalization through the whole molecules. For CPT, the value of bond angle C9-C-N is 179.98°.

In all cases, the atoms in the R group are coplanar with the corresponding benzene ring, which indicates that there are only two planar (thiophene ring and benzene ring) within each of the title compounds. The values of dihedral angles between the thiophene and benzene rings are 29.50°-35.73°. The angles calculated at B3LYP/6-311++G(d,p) level have higher values than that calculated at B3LYP/6-31G(d) level. Nonetheless, the two calculated levels represent the same tendency for the three compounds. FPT has the biggest values of dihedral angle while NPT has the smallest, which indicates that NPT has the highest π-conjugated structure (Haddon 2001).

### 3.2 Vibrational frequency

**Figure 2:**
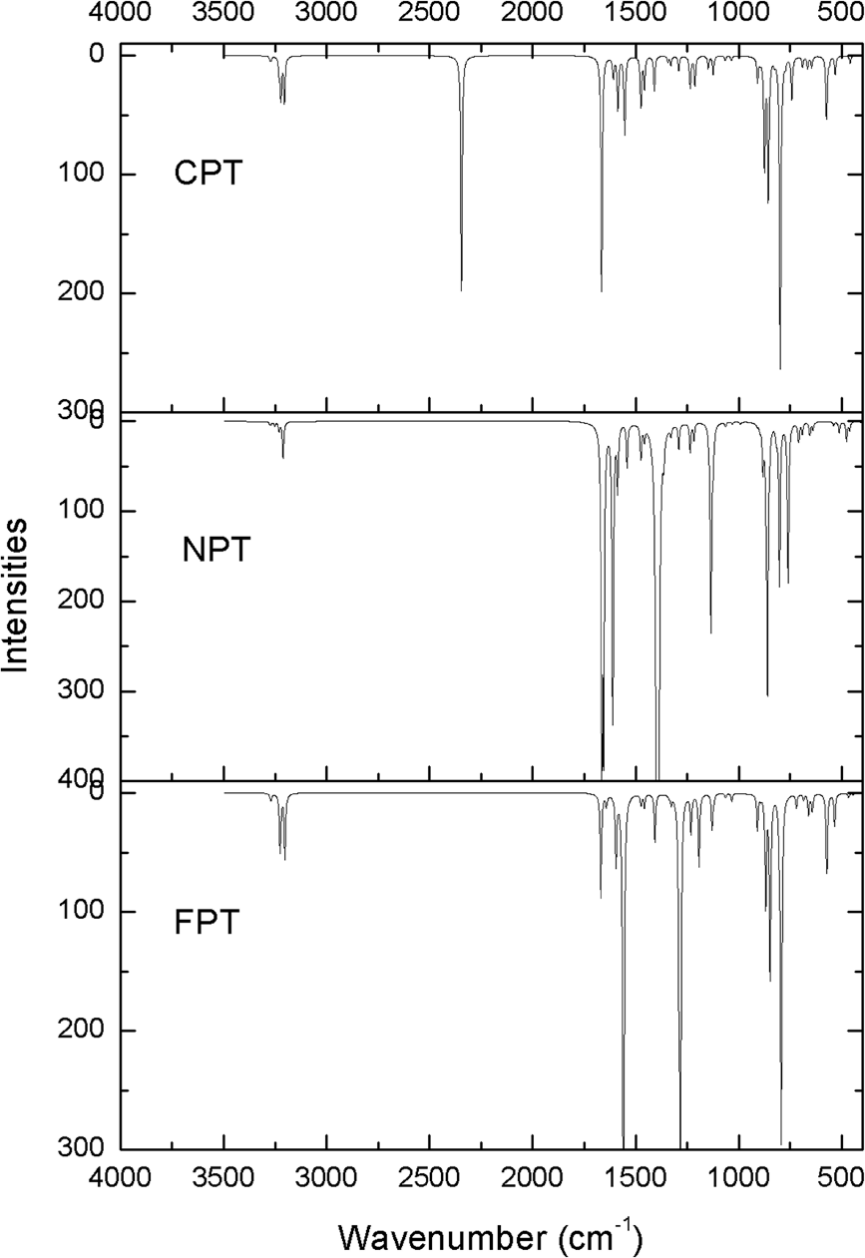
**The theoretically FT-IR spectrum of the title compounds by B3LYP/6-31G(d) methods.**

**Figure 3:**
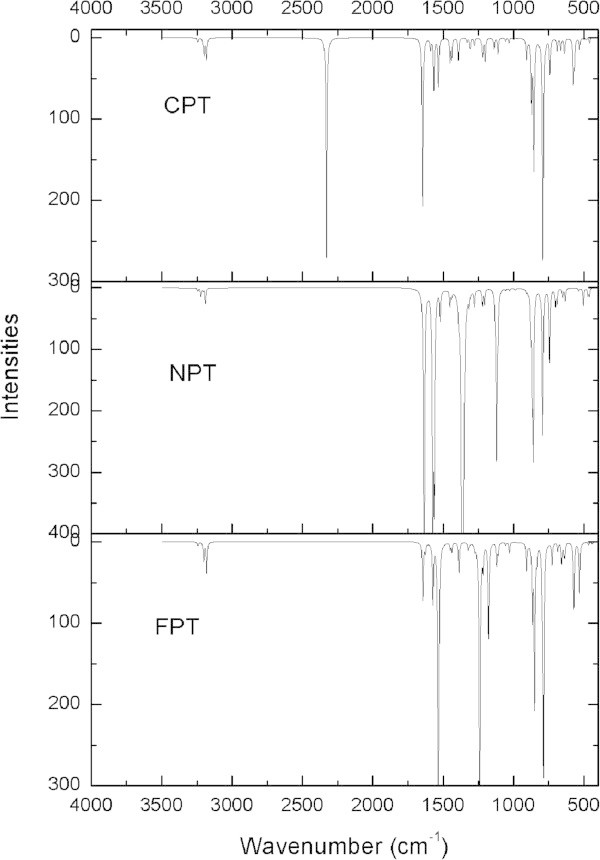
**The theoretically FT-IR spectrum of the title compounds by B3LYP/6-311++G(d,p) methods.**

Vibrational frequencies were calculated by B3LYP/6-31G(d) and B3LYP/6-311++G(d,p) methods. Tables 2, 3, 4 presents the calculated vibrational frequencies over the range 4000–400 cm^−1^ of the title compounds studied. Inclusion of electron correlation in density functional theory to a certain extent makes the frequency values closer to the experimental vibrational frequencies. According to the data in Tables 2, 3, 4, the frequencies values calculated with 6-311++G(d,p) basis set are smaller than that with 6-31G(d) basis set. The following discussions are being done with the results at DFT level calculation with 6-311++G(d,p) basis set for a higher accuracy. Calculated IR intensities help us to distinguish and more precisely assign those fundamentals which are close in frequency (Li et al. 2011). The theoretical FT-IR spectra calculated at B3LYP/6-31G(d) and B3LYP/6-311++G(d,p) levels are shown in Figures 2 and 3, respectively.

**Table 2:** **Calculated vibrational frequencies (cm** ^**−1**^ **) for FPT**

Assignments	FPT	
	6-31G(d)	6-311++G(d.p)
ν(C-H)th	3273(0.96)	3246(0.67)
ν(C-H)th	3269(1.24)	3242(1.02)
ν(C-H)th, ν(C-H)ph	3226(5.40)	3203(2.29)
ν(C-H)th, ν(C-H)ph	3224(5.98)	3201(2.84)
ν(C-H)th, ν(C-H)ph	3223(4.14)	3200(2.26)
ν(C-H)th, ν(C-H)ph	3202(6.33)	3180(3.54)
ν(C-H)ph	3201(9.83)	3179(7.59)
ν(C-C)ph	1669(25.52)	1645(20.75)
ν(C-C)ph	1641(3.02)	1627(3.30)
ν(C-C)ph, ν(C-C)th	1595(16.96)	1573(20.98)
ν(C-C)ph, ν(C-C)th	1560(122.96)	1535(130.98)
ν(C-C)ph, ν(C-C)th	1475(2.83)	1451(2.32)
ν(C-C)ph, ν(C-C)th	1458(3.50)	1439(3.37)
ν(C-C)ph, ν(C-C)th	1406(11.87)	1388(10.73)
ν(C-C)ph, δ(C-H)ip-th	1339(0.63)	1323(2.50)
δ(C-H)ip-th, δ(C-H)ip-ph	1328(2.20)	1314(1.01)
ν(C-C), ν(C-F), δ(C-H)ip-th	1290(41.22)	1275(2.06)
ν(C-F), δ(C-H)ip-th, δ(C-H)ip-ph	1283(81.11)	1241(138.39)
δ(C-H)ip-th, δ(C-H)ip-ph	1232(9.39)	1217(7.52)
δ(C-H)ip-ph	1194(17.64)	1179(34.33)
δ(C-H)ip-ph, δ(C-H)ip-th	1130(8.61)	1119(8,16)
δ(C-H)ip-th, δ(C-H)ip-ph	1122(2.00)	1109(3.73)
δ(C-H)ip-th, δ(C-H)ip-ph	1065(1.07)	1057(1.21)
α*(*ring*)*ph, δ(C-H)ip-ph	1034(2.00)	1030(4.03)
δ(C-H)opp-ph	959(0.23)	973(0.25)
δ(C-H)opp-ph	953(0.03)	956(0.09)
α*(*ring*)*th, δ(C-H)opp-th, α*(*ring*)*ph	911(8.64)	909(9.70)
δ(C-H)opp-th	895(0.72)	895(0.60)
δ(C-H)opp-ph, ν(C-S), δ(C-H)opp-th	871(26.90)	867(25.59)
δ(C-H)opp-ph, δ(C-H)opp-th	849(44.62)	851(58.48)
α*(*ring*)*th, δ(C-H)opp-ph, α*(*ring*)*ph	844(1.46)	835(4.97)
δ(C-H)opp-ph	831(0.41)	826(0.16)
α(ring)th, α(ring)ph, δ(C-H)opp-ph, δ(C-H)opp-th	804(7.14)	797(13.20)
δ(C-H)opp-ph, δ(C-H)opp-th	793(84.60)	788(82.73)
δ(C-H)opp-ph, δ(C-H)opp-th, Φ(ring)ph	720(3.48)	727(7.80)
δ(C-H)opp-ph	685(1.49)	687(3.06)
δ(C-H)opp-ph, Φ(ring)ph, Φ(ring)th	661(5.22)	659(7.52)
Φ(ring)ph, Φ(ring)th	645(3.39)	644(3.97)
Φ(ring)ph, Φ(ring)th	641(1.76)	636(4.69)
α*(*ring*)*th, α*(*ring*)*ph	573(19.72)	573(23.87)
Φ(ring)ph, Φ(ring)th, δ(C-H)opp-ph	535(8.08)	532(18.09)
Φ(ring)th	467(1.03)	468(0.97)
Φ(ring)ph, Φ(ring)th	444(0.42)	442(0.69)
Φ(ring)ph	426(0.09)	426(0.11)

The numbers in the parentheses correspond to the IR intensities. α: planar ring deformation, Φ: non-planar deformation, ν: stretching, δ: bending, ph: benzene, th: thiophene.

**Table 3:** **Calculated vibrational frequencies (cm** ^**−1**^ **) for NPT**

Assignments	NPT	
	6-31G(d)	6-311++G(d.p)
ν(C-H)th	3275(0.75)	3248(0.80)
ν(C-H)th	3271(0.68)	3243(1.01)
ν(C-H)ph	3252(1.02)	3222(3.22)
ν(C-H)ph	3251(0.48)	3221(0.75)
ν(C-H)th, ν(C-H)ph	3230(2.93)	3207(1.42)
ν(C-H)th, ν(C-H)ph	3212(5.72)	3190(3.63)
ν(C-H)ph	3210(6.44)	3188(3.92)
ν(C-C)ph, ν(N-O)	1665(105.58)	1637(68.31)
ν(C-C)ph	1655(96.36)	1635(101.67)
ν(C-C)ph, ν(C-C)th, ν(N-O)	1611(96.07)	1574(144.85)
ν(C-C)th	1588(20.32)	1564(88.76)
ν(C-C)ph, ν(C-C)th	1541(13.83)	1523(13.80)
ν(C-C)ph, ν(C-C)th	1475(10.72)	1453(7.25)
ν(C-C)ph, ν(C-C)th	1458(4.38)	1440(3.14)
ν(C-C)ph, ν(C-C)th	1407(5.29)	1389(6.69)
ν(C-N)	1393(554.93)	1363(593.08)
ν(C-C)ph	1363(7.22)	1346(10.99)
δ(C-H)ip-ph	1329(2.98)	1316(5.55)
δ(C-H)ip-th, δ(C-H)ip-ph	1291(8.10)	1279(7.66)
δ(C-H)ip-th	1236(9.45)	1221(7.50)
δ(C-H)ip-ph	1218(5.70)	1206(6.98)
δ(C-H)ip-ph	1140(7.72)	1131(8.01)
δ(C-H)ip-th, δ(C-H)ip-ph	1135(64.27)	1121(78.97)
δ(C-H)ip-th	1124(10.75)	1110(18.81)
α*(*ring*)*th, δ(C-H)ip-th, δ(C-H)ip-ph	1065(1.34)	1056(1.01)
α*(*ring*)*ph	1033(0.61)	1029(1.26)
δ(C-H)opp-ph	993(0.66)	998(0.08)
δ(C-H)opp-ph	983(0.09)	987(0.99)
α*(*ring*)*th	912(0.88)	910(1.42)
δ(C-H)opp-ph, δ(C-H)opp-th	898(1.60)	898(1.33)
δ(C-H)opp-ph, δ(C-H)opp-th,	884(13.61)	879(13.51)
δ(C-H)opp-ph, ν(C-S), δ(C-H)opp-th	870(15.92)	869(44.51)
α(ring)th, α(ring)ph, δ(C-H)opp-ph, δ(C-H)opp-th, δ(N-O)	862(84.49)	861(72.40)
δ(C-H)opp-ph	851(4.91)	847(1.69)
δ(C-H)opp-ph, α(ring)th	817(4.24)	812(1.70)
δ(C-H)opp-th, δ(C-H)opp-ph, Φ(ring)ph, Φ(ring)th	802(52.29)	793(68.72)
δ(C-H)opp-th, δ(C-H)opp-ph, Φ(ring)ph, δ(C-N)	760(51.52)	745(35.05)
α*(*ring*)*th, α*(*ring*)*ph	716(0.55)	717(0.25)
Φ(ring)ph, δ(C-H)opp-th	709(6.11)	703(7.85)
δ(C-H)opp-th	691(3.68)	691(6.69)
Φ(ring)ph, Φ(ring)th, δ(C-H)opp-th	655(4.22)	652(4.30)
Φ(ring)ph, Φ(ring)th, δ(C-H)opp-th	639(2.62)	634(5.91)
Φ(ring)ph, Φ(ring)th	630(0.14)	630(0.12)
δ(C-N)	540(1.26)	538(1.55)
Φ(ring)ph	512(3/56)	505(8.19)
ν(ph-NO_2_)	477(6.27)	473(3.64)
Φ(ring)th	462(2.64)	463(3.55)
Φ(ring)ph	423(0.05)	420(0.08)
Φ(ring)ph, Φ(ring)th	410(0.26)	406(0.20)

The numbers in the parentheses correspond to the IR intensities. α: planar ring deformation, Φ: non-planar deformation, ν: stretching, δ: bending, ph: benzene, th: thiophene.

**Table 4:** **Calculated vibrational frequencies (cm** ^**−1**^ **) for CPT**

Assignments	CPT	
	6-31G(d)	6-311++G(d.p)
ν(C-H)th	3275(0.77)	3247(0.75)
ν(C-H)th	3270(0.85)	3242(1.02)
ν(C-H)ph, ν(C-H)th	3229(3.65)	3206(1.69)
ν(C-H)ph, ν(C-H)th	3223(0.21)	3199(0.08)
ν(C-H)th, ν(C-H)ph	3221(9.86)	3198(5.73)
ν(C-H)th, ν(C-H)ph	3205(4.14)	3182(2.33)
ν(C-H)ph	3203(7.33)	3181(5.73)
ν(C ≡ N)	2345(57.40)	2328(79.30)
ν(C-C)ph	1665(57.84)	1647(60.69)
ν(C-C)ph, ν(C-C)th	1608(5.00)	1590(3.95)
ν(C-C)ph, ν(C-C)th	1586(13.12)	1567(18.50)
ν(C-C)ph, ν(C-C)th	1553(19.22)	1534(17.38)
ν(C-C)ph, ν(C-C)th	1475(12.16)	1452(8.34)
ν(C-C)ph, ν(C-C)th	1458(7.78)	1440(7.27)
ν(C-C)ph, ν(C-C)th	1409(8.58)	1392(7.99)
δ(C-H)ip-ph	1344(1.37)	1332(1.43)
ν(C-C)ph, δ(C-H)ip-th	1330(2.26)	1309(3.74)
ν(C_ph_-C_th_), δ(C-H)ip-th, δ(C-H)ip-ph	1292(3.60)	1278(2.78)
δ(C-H)ip-th, δ(C-H)ip-ph, ν(C_ph_-CN)	1240(1.60)	1229(0.75)
δ(C-H)ip-th, δ(C-H)ip-ph	1235(7.16)	1220(6.33)
δ(C-H)ip-ph	1213(7.31)	1202(8.18)
δ(C-H)ip-ph	1149(3.00)	1138(3.87)
δ(C-H)ip-th	1124(4.56)	1111(5.71)
α*(*ring*)*th, δ(C-H)ip-th, δ(C-H)ip-ph	1066(1.07)	1057(1.13)
α*(*ring*)*ph, δ(C-H)ip-th, δ(C-H)ip-ph	1037(1.17)	1033(1.85)
δ(C-H)opp-ph	979(0.04)	989(0.09)
δ(C-H)opp-ph	972(0.11)	977(0.17)
α*(*ring*eth*th	911(5.98)	909(7.11)
δ(C-H)opp-th	898(0.92)	898(0.89)
δ(C-H)opp-ph, ν(C-S), δ(C-H)opp-th	877(26.43)	873(24.47)
δ(C-H)opp-ph, δ(C-H)opp-th,	859(32.47)	857(45.62)
δ(C-H)opp-ph	854(4.92)	849(2.68)
α(ring)th, α(ring)ph, δ(C-H)opp-ph, δ(C-H)opp-th	827(1.07)	820(0.99)
δ(C-H)opp-ph, δ(C-H)opp-th	797(75.99)	792(79.51)
α(ring)th, α(ring)ph, δ(C-H)opp-th	773(1.57)	771(1.11)
δ(C-H)opp-th, Φ(ring)ph	742(10.37)	742(12.43)
δ(C-H)opp-th	690(2.66)	691(4.50)
Φ(ring)ph, Φ(ring)th, δ(C-H)opp-th	666(2.98)	665(4.18)
Φ(ring)ph, Φ(ring)th, δ(C-H)opp-th, δ(C-H)opp-ph	647(2.79)	640(4.16)
Φ(ring)th	638(0.22)	637(1.93)
Φ(ring)ph, δ(C-H)opp-ph, δ(C_ph_-CN)	574(15.24)	576(15.05)
Φ(ring)ph, δ(C-H)opp-ph, δ(C_ph_-CN)	561(1.38)	569(7.44)
α(ring)ph, α(ring)th	532(4.37)	532(4.04)
Φ(ring)th, Φ(ring)ph	484(0.05)	485(0.59)
Φ(ring)th	460(1.73)	461(1.83)
Φ(ring)ph	414(0.01)	412(0.01)

The numbers in the parentheses correspond to the IR intensities. α: planar ring deformation, Φ: non-planar deformation, ν: stretching, δ: bending, ph: benzene, th: thiophene.

#### 3.2.1 C-H vibrations

The existence of one or more aromatic rings in a molecule is normally determined from the C-H and C-C = C ring related vibrations. The C-H stretching occurs above 3000 cm^−1^ and is typically exhibited as a multiplicity of weak to moderate bands (Hunt et al. 1987). In the present theoretical study, the FTIR band in the region 3300–3100 cm^−1^ are assigned to aryl C-H stretching vibrations. The C-H in plane bending vibration usually occurs in the region 1400–900 cm^−1^ and is very useful for characterization purpose (Castillo et al. 2012). For FPT, the calculated frequencies 1323, 1314, 1275, 1241, 1217, 1179, 1119, 1057, 1030 cm^−1^ at B3LYP/6-311++G(d,p) are assigned to C-H in plane bending vibrations, which has similar results for NPT and CPT. The C-H out of plane deformations are observed between 1000–500 cm^−1^ for the three title compounds.

#### 3.2.2 C-S vibrations

In our present study, the C-S stretching vibrations are observed at 867 cm^−1^ for FPT, 869 cm^−1^ for NPT and 873 cm^−1^ for CPT, respectively.

#### 3.2.3 C = C stretching

The ring carbon-carbon stretching vibrations occur in the region 1650–1400 cm^−1^. For aromatic six-membered rings, there are two of three bands in this region due to skeletal vibrations, the strongest usually being at about 1500 cm^−1^ (Li et al. 2011). The aromatic C = C stretching is observed at 1645, 1573, 1535 cm^−1^ for FPT while the strongest peak is observed at 1535 cm^−1^. The strongest peaks are observed at 1574 cm^−1^ and 1647 cm^−1^ for NPT and CPT, respectively, which may be due to the electron-withdrawing effect by the nitro and cyano groups.

#### 3.2.4 Ring vibrations

In benzene, six ring deformation frequencies are observed. Three arise from in-plane bending vibrations, corresponding to 1000–600 cm^−1^ mode and the remaining three are derived from the out-of-plane bending vibrations, corresponding to 700–400 cm^−1^ mode of vibrations (Li et al. 2011). For FPT, the α(ring) vibrations are observed at 909, 835, 797, 573 cm^−1^ and Φ(ring) vibrations at 644, 636, 532 cm^−1^.

#### 3.25 C-N vibrations

For NPT, the strong peak at 1363 cm^−1^ is assigned to the C_ph_-N single-bonded stretching. For CPT, the C ≡ N stretching is observed at 2328 cm^−1^.

#### 3.2.6 N-O vibrations

The N-O stretching vibration is observed at 1637 and 1574 cm^−1^ for NPT.

### 3.3 ^13^C and ^1^H NMR studies

The calculated values of ^13^C and ^1^H chemical shifts by B3LYP/6-311++G(d,p) method in the gas phase are summarized in Tables 5 and 6.

**Table 5:** **Calculated** δ**(cal)** ^**13**^ **C chemical shifts of the title compounds**

C	FPT		NPT		CPT	
	6-311++G(d,p)	6-311++G(2d,2p)	6-311++G(d,p)	6-311++G(2d,2p)	6-311++G(d,p)	6-311++G(2d,2p)
2	130.4	129.8	133.9	133.0	132.9	132.2
3	148.1	148.9	146.7	147.6	147.1	148.0
4	130.5	131.1	130.1	130.7	130.0	130.6
5	136.2	136.1	137.2	137.3	137.1	137.1
6	139.4	139.7	149.0	149.4	146.4	146.9
7	133.2	133.2	130.6	130.8	131.2	131.1
8	120.1	120.2	129.6	130.3	138.9	138.8
9	171.1	171.6	153.1	153.6	115.9	116.4
10	119.8	119.9	129.6	130.1	138.8	138.7
11	133.0	133.0	130.5	130.8	131.2	131.1
12^a^	---	---	---		122.1	122.9

^a^C atom in cyano group for CPT.

**Table 6:** **Calculated** δ**(cal)** ^**13**^ **H chemical shifts of the title compounds**

H ^a^	FPT		NPT		CPT	
	6-311++G(d,p)	6-311++G(2d,2p)	6-311++G(d,p)	6-311++G(2d,2p)	6-311++G(d,p)	6-311++G(2d,2p)
2	7.29	7.63	7.57	7.92	7.50	7.85
4	7.30	7.56	7.38	7.67	7.40	7.66
5	7.27	7.68	7.36	7.78	7.33	7.75
7	7.62	7.90	7.67	8.00	7.69	8.00
8	7.19	7.48	8.48	8.94	7.76	8.06
10	7.19	7.49	8.49	8.94	7.77	8.08
11	7.54	7.84	7.57	7.93	7.62	7.94

^a^The number of H are according to the number of the bonded carbon.

(Li and Zhang 2013) calculated the ^13^C and ^1^H chemical shifts of 2-dicyanovinyl-5-(4- methoxyphenyl) thiophene in the gas phase by B3LYP/6-311++G(d,p) and B3LYP/6-311++G(2d,2p) method and the calculated results are good agreement with the experimental ones (Li and Zhang 2013). In order to have a comparison, we extend our study by employing B3LYP/6-311++G(2d,2p) method to calculated the ^13^C and ^1^H chemical shifts in the gas phase. It has been proved that the chemical shifts calculated by B3LYP/6-311++G(2d,2p) method are closer to the experimental values than those calculated by B3LYP/6-311++G(d,p) method (Li et al. 2011). It is noted that all the ^13^C and ^1^H chemical shifts are in there normal values for all the compounds. The ^1^H chemical shifts calculated by B3LYP/6-311++G(2d,2p) method have higher values than those calculated by B3LYP/6-311++G(d,p) method in the present study. For the thiophene ring, C3 has the highest chemical shifts in each compound, which may due to the substituting effects of the benzene moiety. The C9 in the benzene rings have the highest value of chemical shifts for FPT and NPT while that of CPT has the smallest, indicating the effect of the substituted groups at C9.

### 3.4 Mulliken charges

**Figure 4:**
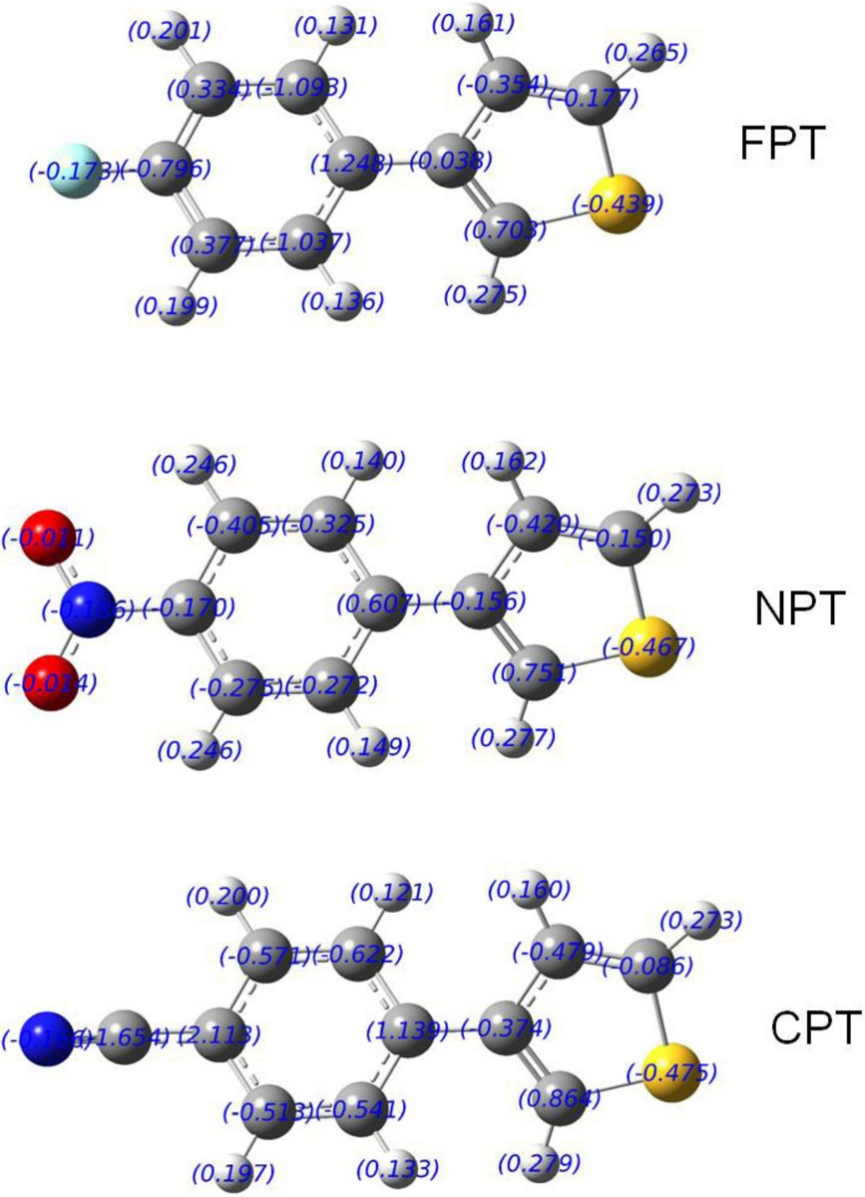
**Atomic charges for optimized geometries of the title compounds at B3LYP/6-311++G(d,p) level.**

**Figure 5:**
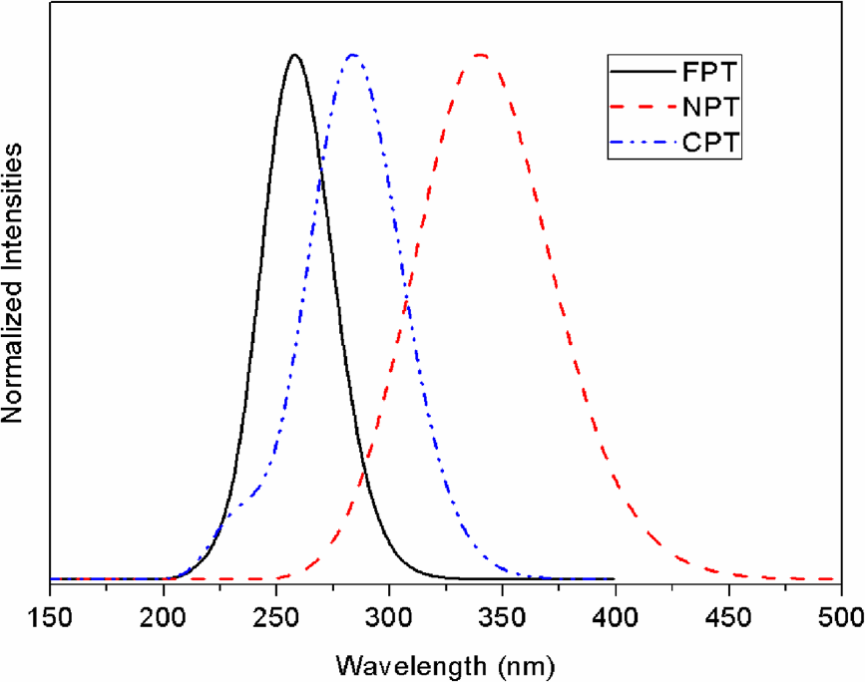
**Predicted UV–vis spectra of the title compounds at B3LYP/6-311++G(d,p) level.**

The atomic charge in the molecules is fundamental to chemistry. Mulliken atomic charges calculated at the B3LYP/6-311++G(d,p) level are shown in Figure 4. It is noted from Figure 5 that the charge distribution of the aromatic skeleton is related with the substituted groups at C9. For example, the charge of C9 atom is −0.796 for FPT, −0.170 for NPT, and 2.113 for CPT. The sum charges of the substituted groups are −0.173 for FPT, −0.211 for NPT, and −1.82 for CPT, which indicates that cyano group has the highest electron-withdrawing effect. The charge values on S atom decrease from −0.439 to −0.475 from FPT to CPT. All the hydrogen atoms have a net positive charge.

Particularly, the charges on H2 and H5 atoms exhibit large positive values (0.275 and 0.265 for FPT, 0.277 and 0.273 for NPT, 0.279 and 0.273 for CPT). The presence of large negative charge on S atom and positive charge on H2 or H5 atom may suggest the formation of intramolecular interactions in the solid states.

### 3.5 Electronic spectra

**Figure 6:**
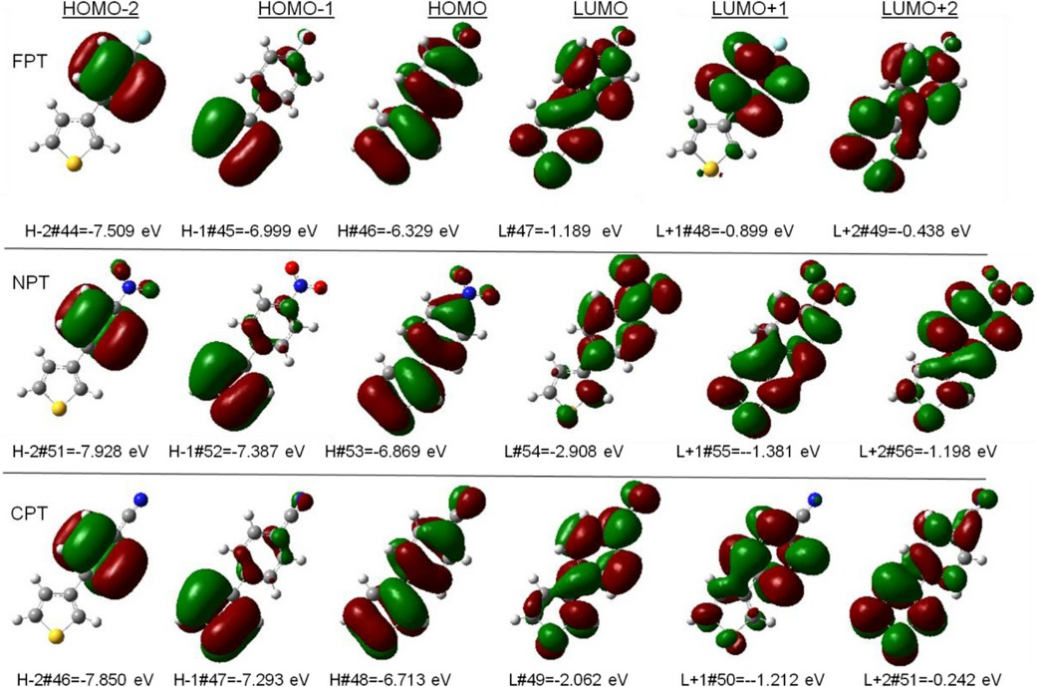
**Frontier molecular orbitals of FPT, NPT and CPT calculated at B3LYP/6-311++G(d,p)//TD- B3LYP/6-311++G(d,p).**

To the best of our knowledge, no experimental UV–vis spectra of the title compounds is reported. Figure 5 display the calculated spectra of the title compounds at B3LYP/6-311++G(d,p) level. Tables 7, 8, 9 list the excitation energies of the Frontier orbitals and oscillator strengths of the optimized ground state geometries. At the B3LYP/6-311++G(d,p) level of theory the excitation bands of the title compounds are composed of mixed HOMO-n → LUMO + m excitations. Figure 6 compares contour plots of three highest occupied and three lowest unoccupied molecular orbitals (H-2 to H, L to L + 2; isovalue 0.02 e/a.u^3^) that give rise to the transitions.

**Table 7:** **B3LYP/6-311++G(d,p) wavelength, excitation energies, and the oscillator strengths for FPT**

State	FPT			
	λ (nm)	eV	***f***	% contribution
S1	265.61	4.668	0.0383	H-2 → L + 0(7%), H-0 → L + 0(27%), H-0 → L + 1(62%)
S2	257.41	4.817	0.2603	H-2 → L + 0(3%), H-0 → L + 0(68%), H-0 → L + 1(24%)
S3	243.84	5.985	0.0002	H-1 → L + 0(50%), H-0 → L + 2(45%)
S4	231.98	5.345	0.0070	H-1 → L + 0(5%), H-0 → L + 2(3%), H-0 → L + 3(78%)
				H-0 → L + 4(8%)
S5	229.23	5.409	0.0104	H-2 → L + 0(8%), H-1 → L + 1(81%), H-0 → L + 3(4%)

**Table 8:** **B3LYP/6-311++G(d,p) wavelength, excitation energies, and the oscillator strengths for NPT**

State	NPT			
	λ (nm)	eV	***f***	% contribution
S1	344.39	3.600	0.3120	H-3 → L + 0(2%), H-0 → L + 0(96%)
S2	329.99	3.757	0.0085	H-3 → L + 0(91%), H-3 → L + 1(2%), H-0 → L + 0(2%)
S3	310.18	3.997	0.0793	H-1 → L + 0(98%)
S4	290.03	4.275	0.0077	H-5 → L + 0(29%), H-2 → L + 0(61%), H-0 → L + 1(2%)
				H-0 → L + 2(4%)
S5	288.72	4.294	0.0042	H-5 → L + 0(66%), H-2 → L + 0(27%)

**Table 9:** **B3LYP/6-311++G(d,p) wavelength, excitation energies, and the oscillator strengths for CPT**

State	CPT			
	λ (nm)	eV	***f***	% contribution
S1	287.11	4.318	0.4520	H-0 → L + 0(94%)
S2	268.77	4.613	0.0590	H-2 → L + 0(10%), H-1 → L + 0(55%), H-0 → L + 1(33%)
S3	265.04	4.678	0.0735	H-2 → L + 0(20%), H-1 → L + 0(38%), H-0 → L + 1(34%)
				H-0 → L + 2(6%)
S4	237.49	5.220	0.0137	H-3 → L + 0(5%), H-3 → L + 1(2%), H-2 → L + 0(21%)
				H-1 → L + 0(3%), H-1 → L + 1(3%), H-1 → L + 2(2%)
				H-0 → L + 1(20%), H-0 → L + 2(40%)
S5	232.34	5.34	0.0512	H-3 → L + 0(6%), H-3 → L + 1(4%), H-2 → L + 0(13%)
				H-1 → L + 1(40%), H-0 → L + 1(5%), H-0 → L + 2(29%)

#### (4-fluorophenyl)thiophene (FPT)

The S1-S5 bands of FPT are calculated at 265, 257, 243, 231, 229 nm. The nature of the strongest absorption band 257 nm (S0 → S2) is dominated by excitations from HOMO-2 → LUMO + 0, HOMO-0 → LUMO + 0, and HOMO-0 → LUMO + 1, which consist of n → π* and π → π* transitions (see Frontier orbitals in Figure 6). The oscillator strength of S0 → S1 band is *f* = 0.0383, and the major excitation is HOMO-0 → LUMO + 1(62%), which is assigned to the n → π* transition. The HOMO-LUMO gap is calculated to be 5.14 eV.

#### (4-nitrophenyl)thiophene (NPT)

The S1-S5 bands of NPT are calculated at 344, 329, 310, 290, 288 nm, which shows red-shifted character compared with FPT. The nature of the strongest absorption band 344 nm (S0 → S1) is dominated by excitations from HOMO-0 → LUMO + 0(94%), which consist of π → π* transitions (see Frontier orbitals in Figure 6). The HOMO-LUMO gap is calculated to be 3.961 eV, which is lower than that of FPT. It is reported that the molecules with nitro group can lower the band gaps, which has potential use in photovoltaic cells (Mikroyannidis et al. 2009).

#### (4-cyanophenyl)thiophene (CPT))

The S1-S5 bands of CPT are calculated at 287, 268, 265, 237, 232 nm, which shows red-shifted character compared with FPT, and blue-shift compared with NPT. The nature of the strongest absorption band 287 nm (S0 → S1) is dominated by excitations from HOMO-0 → LUMO + 0(96%), which consist of n → π* and π → π* transitions (see Frontier orbitals in Figure 6). The HOMO-LUMO gap is calculated to be 4.651 eV, which has the intermediate value among the three compounds.

## 4. Conclusions

In the present work, the optimized molecular structures, vibrational frequencies, NMR chemical shifts, and electronic properties of the three title compounds have been calculated by using B3LYP/6-31G(d), B3LYP/6-311++G(d,p) and TD-B3LYP/6-311++G(d,p) methods. The optimized geometries results show that FPT has the biggest values of dihedral angle while NPT has the smallest, which indicates that NPT has the highest π-conjugated structure. The vibrational frequencies values calculated with 6-311++G(d,p) basis set are smaller than that with 6-31G(d) basis set for all the compounds. For NPT, the strong peak at 1363 cm^−1^ is assigned to the C_ph_-N single-bonded stretching, and the N-O stretching vibration is observed at 1637 and 1574 cm^−1^. For CPT, the C ≡ N stretching is observed at 2328 cm^−1^. The C9 in the benzene rings have the highest value of chemical shifts for FPT and NPT while that of CPT has the smallest, indicating the effect of the substituted groups at C9. CPT shows red-shifted character compared with FPT, and blue-shift compared with NPT in the TD-DFT calculations. In a word, the type of substituted groups at the C9 atom have significant effect on the properties for the 3-(4-phenyl)thiophene derivatives. Poly(3-phenylthiophene) has been used reported for used in supercapacitors. The polymerization of the three title compounds are being studied by our group. We believe that the three title compounds will show good performance in supercapacitors.
